# The Elements of Bacteriological Technique

**Published:** 1903-11

**Authors:** 


THE ELEMENTS OF BACTERIOLOGICAL TECHNIQUE.—A Laboratory Guide for the Medical, Dental and Technical Student. By J. W. H. Eyre, M. S., M. D., Bacteriologist to Guy's Hospital, London. 170 illustrations. 362 pages. Price, $3.00

This is a comprehensive and practically arranged treatise on the subject of bacteriology designed for the use of those desiring to do work of a clinical or investigative nature along this line. The work is logically arranged in outline form, each new phase of the subject being introduced by sufficient explanation to enable the beginner to grasp the principles of the methods used.

Profuse illustrating is aptly depended on to make the text clear, and this, by the way, we regard as a most commendable feature.

On the whole we can heartily recommend the work as coming up to what is claimed for it—an up-to-date treatise on the subject. J. M. L.

